# Lipin1 Is Involved in the Pathogenesis of Diabetic Encephalopathy through the PKD/Limk/Cofilin Signaling Pathway

**DOI:** 10.1155/2020/1723423

**Published:** 2020-10-16

**Authors:** Min Xie, Meijian Wang, Wei Liu, Min Xu, Pan Shang, Dongqing Jiang, Liping Ju, Fei Wu, Aili Sun, Shuyan Yu, Xianghua Zhuang, Shihong Chen

**Affiliations:** ^1^Department of Endocrinology and Metabolism, The Second Hospital, Cheeloo College of Medicine, Shandong University, Jinan 250012, China; ^2^Department of Endocrinology and Metabolism, Binzhou Medical University Hospital, Binzhou 256603, China; ^3^Department of Encephalopathy, The Second Affiliated Hospital of Shandong University of Traditional Chinese Medicine, Jinan 250001, China; ^4^Department of Physiology, School of Basic Medical Sciences, Cheeloo College of Medicine, Shandong University, Jinan 250012, China

## Abstract

Diabetic encephalopathy is a type of central diabetic neuropathy resulting from diabetes mainly manifested as cognitive impairments. However, its underlying pathogenesis and effective treatment strategies remain unclear. In the present study, we investigated the effect of Lipin1, a phosphatidic acid phosphatase enzyme, on the pathogenesis of diabetic encephalopathy. We found that *in vitro*, Lipin1 exerts protective effects on high glucose-induced reductions of PC12 cell viability, while *in vivo*, Lipin1 is downregulated within the CA1 hippocampal region in a type I diabetes rat model. Increased levels of Lipin1 within the CA1 region are accompanied with protective effects including amelioration of dendritic spine and synaptic deficiencies, phosphorylation of the synaptic plasticity-related proteins, LIM kinase 1 (p-limk1) and cofilin, as well as increases in the synthesis of diacylglycerol (DAG), and the expression of phosphorylated protein kinase D (p-PKD). These effects are associated with the rescue of cognitive disorders as shown in this rat model of diabetes. In contrast, knockdown of Lipin1 within the CA1 region enhanced neuronal abnormalities and the genesis of cognitive impairment in rats. These results suggest that Lipin1 may exert neuroprotective effects involving the PKD/Limk/Cofilin signaling pathway and may serve as a potential therapeutic target for diabetic encephalopathy.

## 1. Introduction

Diabetes can result in chronic complications involving multiple systems. In some diabetic patients, varying degrees of cognitive deficits and even dementia may be present. Accordingly, the term “diabetic encephalopathy (DE)” was first proposed in 1950 to describe the cognitive impairments related to diabetes [1]. Such impairments in cognitive function, which can be revealed as declines in learning, attention, and spatial memory, comprise an important basis for the disability and death of these patients. However, the exact pathogenesis of DE is quite complex and remains unclear [2]. Therefore, a more detailed understanding of DE pathogenesis will be required in order to identify new approaches for the prevention and treatment of this condition.

Lipin1, a phosphatidic acid phosphatase enzyme, plays an important role in glycolipid metabolism [3] and participates in triacylglycerol biosynthesis through either a Mg^2+^-dependent phosphatidate phosphatase or as a transcriptional coactivator of genes in the oxidative metabolism of fat [4, 5]. In the muscle, Lipin1 can convert phosphatidic acid (PA) to diacylglycerol (DAG) to further activate protein kinase D (PKD) [6]. In addition, Lipin1 also appears to exert effects within the nervous system as demyelination is observed in Lipin1 mutant mice, effects which appear to be mediated by a PA metabolic disturbance [7].

Previous work within our laboratory has revealed that the occurrence of diabetic peripheral neuropathy (DPN) in rats may be related to a decline in Lipin1 expression, while an upregulation in Lipin1 levels improves nerve conduction velocity and the pathological morphology observed within a diabetes mellitus (DM) rat model [8]. We have also found that cognitive impairments were present in Lpin1^fld^/J mice [9]. Whether Lipin1 may be involved in the pathogenesis of DE and, if so, the potential molecular mechanisms that underlie such effects have not been elucidated. Accordingly, the goal of this report was to address these issues. As an approach to accomplish this goal, we have conducted this study.

## 2. Materials and Methods

### 2.1. Animals

Male Wistar rats (6–8 weeks old) were obtained from the Shandong University Animal Center. All animal experiments were approved by the Animal Care and Use Committee of Shandong University and were conducted in accordance with the National Institutes of Health guidelines for the care and use of laboratory animals. Rats were maintained on a 12 h light/dark cycle at room temperature of 24 ± 2°C in the animal laboratory of the Shandong University.

### 2.2. *In Vivo* Diabetes Model

For the *in vivo* model of diabetes, streptozocin (STZ, Solarbio, China), which was freshly prepared in cold 0.1 M citrate buffer (pH 4.5, Solarbio, China), was injected intraperitoneally (i.p.) into rats using a single dose of 60 mg/kg body weight. Rats in the control group were injected with an equivalent dose of citrate buffer. Three days after this STZ injection, fasting blood glucose (FBG) levels (with a minimal fasting period of 8 h) were determined. Levels > 11.1 mM were established as the diagnostic criteria for T1DM in this rat model. Blood glucose values were monitored as achieved from samples collected from the tail veins of the rats. Blood glucose levels and body weights were determined at 1, 2, 4, 8, and 12 weeks after the STZ injection. All animal experiments were conducted in the Department of Physiology of the School of Basic Medical Sciences at Shandong University.

### 2.3. *In Vivo* Central Nervous System Treatment

Lentiviral up- and downregulation of the Lipin1 and the blank vectors were constructed and encoded with the green fluorescent protein sequence (Jikai Gene Chemical Technology Co., Ltd.): Lv-Lipin1 (1 × 10^9^ TU/mL), Lv-Lipin1-Con (2 × 10^9^ TU/mL), Lv-Lipin1ShRNA (7 × 10^8^ TU/mL), and Lv-Lipin1ShRNA-Con (1 × 10^9^ TU/mL). The sequence of Lipin1ShRNA was caGCGAGTCTTCAGACACTTT. The sequence of Lipin1ShRNA-Con is TTCTCCGAACGTGTCACGT.

The rats were anesthetized with sodium pentobarbital (40 mg/kg, i.p.) and placed in a stereotaxic frame (Stoelting, USA). The Lv-Lipin1ShRNA and blank vector was injected into WT rats (WT+Lv-Lipin1ShRNA and WT+Lv-Con groups). The Lv-Lipin1 and blank vector was injected into DE rats (DE+Lv-Lipin1 and DE+Lv-Con groups). These rats would then be tested for behavioral responses as described below. The lentovirus (Lv) injection was administered bilaterally to the hippocampal CA1 region (A/P, −3.24; L, ±2.5; D/V, −2.45 mm relative to bregma). A total of 10 *μ*L was administered (5 *μ*L per side of hippocampus) at a flow rate of 0.15 *μ*L/min with use of a 5 *μ*L microsampler (Gaoge, Shanghai, China). The needle remained at the injection site for 10 min and was then gently retracted. The wound was then sterilized and sutured. At 14 days after injection, the rats were anesthetized and slowly perfused with saline and 4% paraformaldehyde (PFA). The brain was carefully removed and placed in 4% PFA at 4°C overnight before undergoing graded dehydration. Frozen brain samples were cut into 30 *μ*m thick consecutive coronal sections and stored at −20°C for the subsequent experiments.

### 2.4. Behavioral Tests

#### 2.4.1. Open Field Test

In the open field test, the rats' movement within a novel environment was recorded, which can serve as a means to evaluate their activity and anxiety. Each rat was placed in the center of the open field box (100 × 100 × 50 cm) at the beginning of the test [10]. The floor of the apparatus was divided into 25 squares. The motion trajectory of each rat was recorded for 5 min using a computerized video system (SMART, Panlab, Spain). The time spent in the central squares and the number of crossings of the squares were then used to assess the degree of anxiety, with maintenance in the center squares and limited crossings indicating high levels of anxiety. After each open field test, the cage was carefully cleaned with 75% ethanol to eliminate any cues which could affect the behavioral responses of the following rats.

#### 2.4.2. Novel Object Recognition (NOR) Test

The NOR test provides a means to evaluate the learning and memory ability of the rats. The task was divided into an adaptation, training, and testing period [11, 12]. For the adaption period, the rats were placed into the apparatus at the same position and allowed to move freely for 5 min. At 24 h after this adaption period, the training period was initiated. During the training period, two identical objects (A1 and A2) were placed symmetrically in the left and right corners of the test box. The time and frequency of exploratory behavior directed to the objects were recorded for 5 min. After this training period, the rats were returned to their original home cage for a 1 h period. During the test period, the rats were again placed in the test apparatus, which now contained a (novel) B object in place of the A2 object. Similar to that of the training period, time and frequency of exploratory behavior directed to the objects (A1 and B) were recorded for 5 min.

The criteria required for defining this as exploratory behavior directed to the objects consisted of direct contact with the object and/or sniffing or licking the object or being located within 2–3 cm from the object. After each rat completed the test, the box and objects were wiped with 75% alcohol to eliminate any cues which could affect the behavioral responses of the following rats. A cognitive function score was defined by a discrimination index (DI) = (novel object  exploration  time − familiar  object  exploration  time)/total exploration time.

#### 2.4.3. Morris Water Maze (MWM)

The MWM is a classic experiment for assessing spatial learning and memory function in rats [13]. A circular chamber (120 cm in diameter and 60 cm in height) was divided into four equal quadrants and contained water with black food dye to ensure that the water was opaque. The depth of the water was 40 cm, and the temperature was maintained at 22 ± 2°C. A platform (13 cm in diameter and 38 cm in height) was placed in the center of quadrant three and was submerged to 2 cm underwater. Markers were positioned around the pool to help the rats locate the platform. The test consisted of four days of place navigation tests with four trials per day and one day of a spatial probe test. Rats within the various experimental treatments (as described above) were randomly placed in quadrant three and allowed to locate the platform within 60 s during the first four days of the navigation tests. If the rats failed to locate the platform, they were gently guided to the platform and allowed to remain on the platform for 10 s. Escape latencies (60 s maximum) and times required to locate the platform were recorded. On the fifth day, the platform was removed, and each rat was allowed to swim freely for 60 s. The time expended crossing over the area where the platform was located was recorded. All data and motion trajectories were recorded using a computerized video system (SMART, Panlab, Spain).

#### 2.4.4. *In Vitro* Diabetes Model

For the *in vitro* diabetes model experiments, the PC12 (rat adrenal pheochromocytoma) cell line, purchased from the Shanghai Cell Bank of Chinese Academy of Science (Shanghai, China), was used. The cells were cultured in 25 mM Dulbecco's modified Eagle's medium under normal glucose (NG) concentrations (DMEM, Invitrogen, Carlsbad, CA, USA) with 1% penicillin/streptomycin (HyClone, USA) and 10% fetal bovine serum (Lonsera, China) at 37°C in a humidified incubator containing 5% CO_2_. The medium was changed every two days. The cells were grown to approximately 80%–90% confluency. All cells were plated at an appropriate density in accordance with the needs of the experimental design.

### 2.5. CCK-8 Assay

A total of 4000 PC12 cells were seeded into 96-well plates and cultured for 12 h in complete medium. The cells were then cultured in DMEM containing 25, 50, 75, 100, 125, or 150 mM glucose for 24 or 48 h. In addition, equal concentrations of mannitol were used as osmolarity controls to exclude the effect of osmotic pressure. Changes in cell viability were determined with the use of a cell counting kit-8 (Dojindo, Japan) according to the manufacturers' instructions. Cell proliferation was determined at 450 nm with the use of a microplate reader.

### 2.6. Virus Infection

The PC12 cells were seeded into 96-well plates at a density of 4000 cells/well and cultured for 24 h. Subsequently, lentiviral vectors (LVs; multiplicity of infection = 100), and a transfection enhancer, HitransG A (Jikai Gene Chemical Technology Co., Ltd.) were added to the cultured cells. The culture solution was changed after 8 h.

### 2.7. Quantitative Real-Time Polymerase Chain Reaction (qRT-PCR)

RNA from PC12 cells and tissues was extracted using the TRIzol reagent (Invitrogen, Carlsbad, CA, USA), while cDNA was generated using the primeScript RT reagent kit (TaKaRa, Otsu, Japan) in accordance with the manufacturers' instructions. qRT-PCR was performed using the SYBR® Green Dye kit (TaKaRa, Otsu, Japan) and the Bio-Rad CFX96 system. Relative gene expression levels were calculated using the classic 2^−ΔΔCt^ method [14]. The primer sequences were as follows: Lipin-1 forward TATGACACGGCTTGTTCC, reverse GTGGCTGCCCTGTATTTC; *β*-actin forward CCTAGACTTCGAGCAAGAGA, reverse GGAAGGAAGGCTGGAAGA (Generay, Shanghai, China).

### 2.8. Western Blot

Rats were euthanized while under anesthesia, and hippocampal CA1 tissues were immediately removed and stored at −80°C until processed for protein extraction. Cellular and the tissue proteins were extracted on ice using cold RIPA buffer (Beyotime, Shanghai, China) supplemented with a protease inhibitor mixture and phenylmethylsulfonyl fluoride (Beyotime, Shanghai, China). The samples were subjected to three episodes of ultrasound for 10 s each, if necessary, and then centrifuged at 12,000 rpm for 20 min at 4°C. The supernatant was heated at 95°C for 5 min and retained for Western blot analyses. Total protein concentrations were determined using the BCA kit (Beyotime, Shanghai, China). Equal amounts of proteins were fractionated using 8% or 12% sodium dodecyl sulfate polyacrylamide gel electrophoresis and transferred onto PVDF membranes. The membranes were blocked with 5% bovine serum albumin (BSA) for 1 h at room temperature and incubated overnight at 4°C with the primary antibodies, anti-Lipin1 (1 : 250, ab181389), anti-tubulin (1 : 5,000, abway), anti-glyceraldehyde-3-phosphate dehydrogenase (GAPDH; 1 : 5,000, proteintech), anti-Ppkd (1 : 500, Ser744/748, CST2054), anti-Pssh1 (1 : 250, pSer978, Novus, NBP1-50636), anti-Limkinase1 (1 : 500, phosphoT508, ab38508), anti-Pcofilin (1 : 250, phosphoS3, ab12866), and anti-Synaptophysin (1 : 7,500, ab32127). The membranes were then washed three times with TBST for 5 min each and incubated with the secondary antibodies conjugated with HRP (1 : 5000) for 1 h at room temperature followed by 3 washings with TBST for 10 min each. Detection was performed using an enhanced chemiluminescence detection kit (Merck Millipore, Billerica, MA, USA). Intensities of the blots were quantified using Image J software.

### 2.9. Transmission Electron Microscopy

The hippocampal CA1 region (1 mm^3^) was collected, fixed with 2% glutaraldehyde, dehydrated with use of an ethanol gradient, embedded, and sectioned. Samples were then stained with uranyl acetate followed by lead citrate on the copper grids. Neurons within these hippocampal CA1 regions were then observed under a transmission electron microscope (Philips Tecnai 20 U-Twin, Holland).

### 2.10. Golgi Stain

Brains were impregnated using the Golgi stain kit (Servicebio, Wuhan, China) in accordance with the manufacturers' instructions. Coronal sections (100 *μ*m thickness) from the CA1 hippocampal region were analyzed from microscopic images as processed using Fiji software.

### 2.11. Enzyme-Linked Immunosorbent Assay (ELISA)

Diacylglycerol concentrations within the hippocampal CA1 region were detected using ELISA kits (J&L Biological, China) with reference to the supplier's description.

### 2.12. Statistical Analysis

All data are presented as means ± SEMs and were analyzed using the IBM SPSS Statistics 22.0 program. Comparisons between groups were performed using unpaired *t*-tests, while comparisons involving multiple groups were analyzed using one-way ANOVA. A *P* value < 0.05 was required for results to be considered as statistically significant.

## 3. Results

### 3.1. Lipin1 Enhances Viability in PC12 Cells Exposed to High Glucose Concentrations

**Figure 1:**
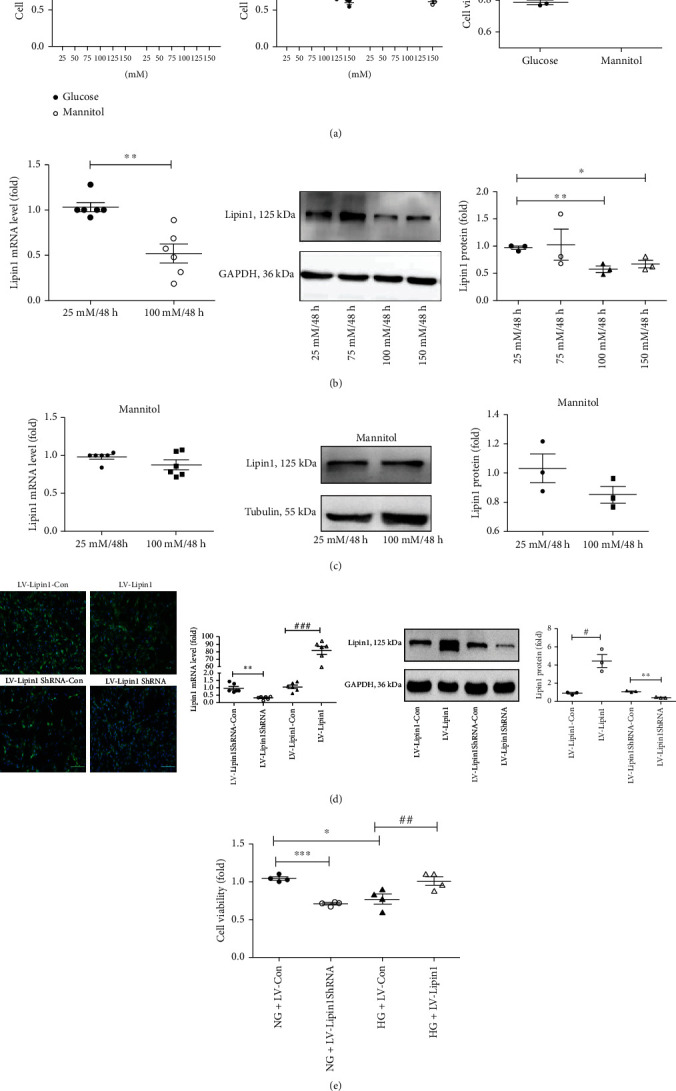
Changes in PC12 cell viability in response to high glucose toxicity are related to Lipin1. (a) Cell viability after 24 or 48 h of treatment with different concentrations of glucose and mannitol, and the cells were incubated in the same concentrations (100 mM) of glucose and mannitol for 48 h. The cell viability of each group was standardized to that of the control group. (b) Incubated with glucose, lipin1 mRNA and protein expressions as determined using qPCR and Western blot. (c) Incubated with mannitol, lipin1 mRNA and protein expressions as determined using qPCR and Western blot. ^∗^*P* < 0.05, ^∗∗^*P* < 0.01, versus 25 mM/48 h. (d) Cells infected with Lv-Lipin1, Lv-Lipin1-Con, Lv-Lipin1ShRNA, and Lv-Lipin1ShRNA-Con for three days. Nucleus were stained blue with 4′,6-diamidine-2-phenylidole dihydrochloride (DAPI). Infection efficiencies were determined using fluorescence microscopy while postinfection Lipin1 mRNA and protein expressions were determined using qPCR and Western blot analysis. ^∗^*P* < 0.05, ^∗∗^*P* < 0.01, versus LV-Lipin1ShRNA–Con group; ^#^*P* < 0.05, ^###^*P* < 0.001 versus LV-Lipin1-Con group. (e) Determinations of viability in cells transfected with different viruses under different treatment conditions. ^∗^*P* < 0.05, ^∗∗∗^*P* < 0.001 versus NG+LV-Con group; ^##^*P* < 0.01, versus HG+LV-Con goup. Scale bar in (d) = 100 *μ*m. Data are expressed as means ± SEMs. Results are representative of three independent experiments.

PC12 cells were cultured for either 24 or 48 h under different concentrations of glucose and mannitol (25-150 mM, with 25 mM serving as the control concentration), and cell viability was assessed using the CCK-8 assay. As shown in Figure 1(a), there was a concentration-dependent decrease in cell viability in response to these treatments. Cells cultured with glucose and mannitol concentrations of 150 mM for 24 or 48 h and 125 mM for 48 h showed statistically significant decreases in viability. Cell viability is decreased when cultured with glucose in 100 mM/48 h compared to mannitol in the same conditions (*P* < 0.05). To eliminate potential cytotoxic effects of increased osmolarity, the 125 and 150 mM glucose concentrations were excluded from further analyses. As decreases in cell viability with 100 mM/48 h (*P* < 0.01) were more evident than that obtained with 75 mM/48 h (*P* < 0.05), the 100 mM/48 h condition was chosen for use as the high glucose (HG) condition in subsequent experiments.

The mRNA (*P* < 0.01) and protein (*P* < 0.01) levels of Lipin1 were significantly decreased in the HG condition (Figure 1(b)). Cultured with mannitol, the mRNA and protein levels of Lipin1 were no statistic difference at the condition of 100 mm/48 h compared to control (Figure 1(c)). Therefore, given these results indicating that Lipin1 was associated with these reductions in cell viability from HG, a LV for Lipin1 upregulation and downregulation was constructed for use in transfection within these PC12 cells to further clarify this relationship between Lipin1 and cell viability. Infection efficiency was visualized using fluorescence microscopy, with the result that >90% of the cells were infected. This infection efficiency was further verified using qPCR and Western blot analysis (Figure 1(d)). Levels of Lipin1 mRNA in the LV-Lipin1 group were significantly increased as compared with those in the LV-Lipin1-Con group (*P* < 0.001), whereas mRNA levels of Lipin1 in the LV-Lipin1ShRNA group were significantly decreased as compared with those in the LV-Lipin1ShRNA-Con group (*P* < 0.05). Similar changes in Lipin1 protein levels as that observed for mRNA were obtained in these conditions.

Cells infected with Lv-Lipin1-Con and Lv–Lipin1 were subjected to HG conditions (HG+Lv-Con and HG+Lv-Lipin1 groups) to assess the role of Lipin1 in glucose-induced injury. Cells infected with Lv–Lipin1ShRNA-Con and Lv-Lipin1ShRNA were treated with the (control) 25 mM glucose and mannitol concentration (NG+Lv-Con and NG+Lv-Lipin1ShRNA group) (Figure 1(e)). Cell viability within the HG+Lv-Lipin1 group was significantly increased as compared with that obtained in the HG+Lv-Con group (*P* < 0.01), while cell viability within the NG+Lv-Lipin1ShRNA group was significantly decreased as compared with that of the NG+Lv-Con group (*P* < 0.001). Lipin1 overexpression attenuated the HG-induced decrease in the viability of PC12 cells, while the silencing of Lipin1 led to a decrease in the viability of PC12 cells as observed under NG condition. These results suggest that Lipin1 may be related to the changes in PC12 cell viability as observed in response to HG exposure.

### 3.2. Lipin1 Is Reduced in the Hippocampal CA1 Region of Diabetic Rats

**Figure 2:**
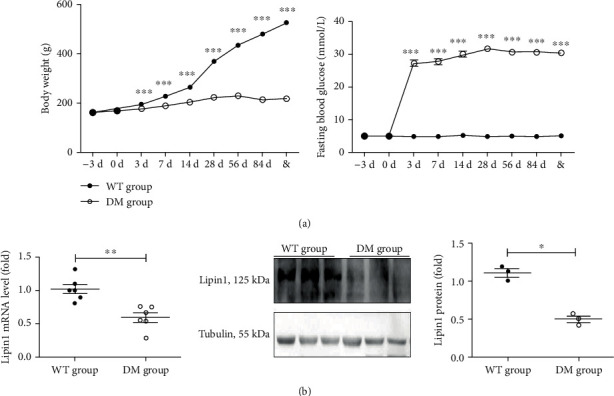
Changes in Lipin1 within the hippocampal CA1 region of diabetic rats. (a) Changes in body weights and blood glucose levels in rats after STZ injection, indicating the induction of a diabetic condition (*n* = 30 animals/group). (b) Expression of Lipin1 levels in the hippocampal CA1 region as determined using qPCR (*n* = 6 animals/group) and Western blot analysis (*n* = 3 animals/group). Data are expressed as the means ± SEMs,^∗^*P* < 0.05, ^∗∗∗^*P* < 0.01, ^∗∗∗^*P* < 0.001 versus wild type group and & means as determined at 14 days after STZ injection.

From the third day after STZ injection, body weights were significantly decreased (*P* < 0.001), and FBG levels significantly increased (*P* < 0.001) in this rat model of diabetes as compared with that of the wild-type (WT) group (Figure 2(a)). These results suggest that a diabetic condition was successfully achieved in these rats. Associated with this diabetic condition were significant decreases in mRNA (*P* < 0.01) and protein (*P* < 0.05) levels of Lipin1 within the hippocampal CA1 region in these diabetic rats as compared with controls (Figure 2(b)).

### 3.3. Lipin1 Content within the CA1 Region Affects Cognitive Functions in Rats

**Figure 3:**
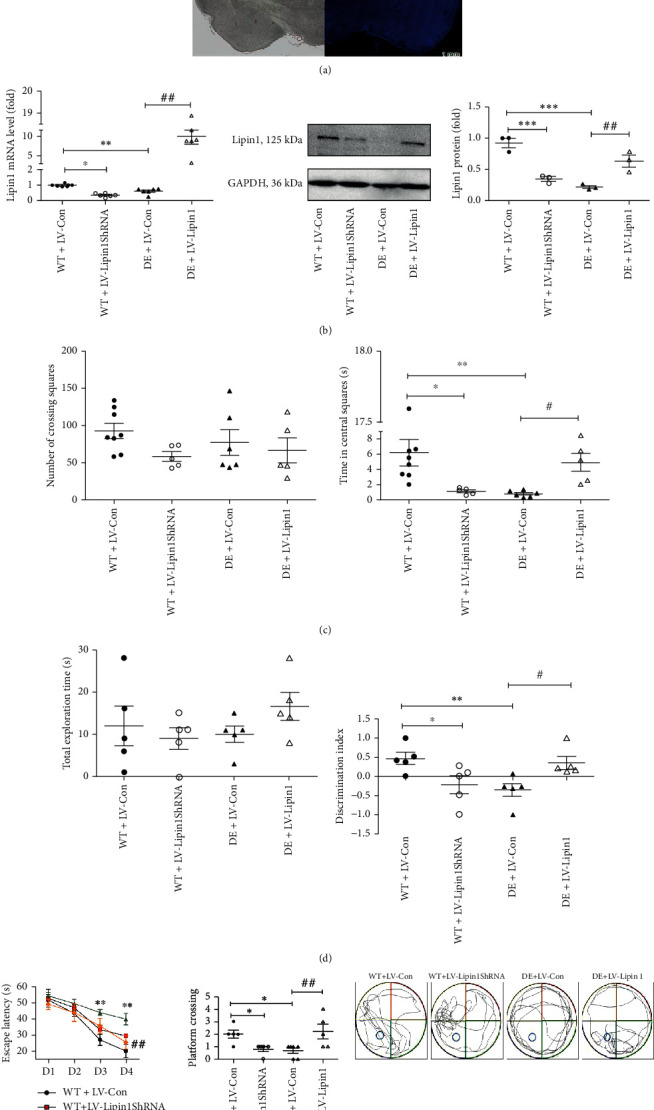
Results of behavioral tests in rats in response to LV injection. (a) Illustration of the viral infusion of LV-Lipin1. (b) The Lipin1 content in the CA1 region after LV injection as determined with use of qPCR and Western blot analysis. (c) Open field test. The number of squares crossed indicates general locomotor activity while time in central area indicates the amount of exploration. (d) Novel object recognition test. The total exploration time and discrimination index were analyzed. (e) Morris water maze test. The escape latencies on different training days and the number of platform crossings were determined. Movements of the rats were also recorded using tracking software. Data represent the means ± SEMs, *n* = 5–8 animals per group. ^∗^*P* < 0.05, ^∗∗^*P* < 0.01, ^∗∗∗^*P* < 0.001 versus WT+LV-Con group; ^#^*P* < 0.05, ^##^*P* < 0.01 versus DE+LV-Con group. Scale bar in (a) = 1 mm.

Within the diabetic rats, those showing DE as revealed by their behavioral test performance were selected to assess whether Lipin1 was related to cognitive function, as well as to determine whether Lipin1 content within the CA1 region was changed in response to a stereotactic injection of the virus into this site. Two complementary approaches to examine this issue involved LV-Lipin1ShRNA injection into WT rats to reduce Lipin1 and LV-Lipin1 injection into DE rats to increase Lipin1. The blank vector was injected as a control (Figure 3(a)). Infection efficiencies were estimated with the use of qPCR and Western blot analysis (Figure 3(b)). The mRNA levels of Lipin1 in the WT+LV-Lipin1ShRNA (*P* < 0.05) and DE+LV-Con (*P* < 0.01) groups were significantly decreased as compared with those in the WT+LV-Con group, whereas these Lipin1 mRNA levels in the DE+LV-Lipin1 group were significantly increased as compared with those in the DE+LV-Con group (*P* < 0.01). Similar changes in Lipin1 protein levels were observed in these conditions.

Behavioral tests were performed at 14 days after stereotactic injection of the virus to assess the effects of the changes in Lipin1. In the open field test, no differences were obtained in the number of crossings of the squares among the groups (*P* > 0.05, Figure 3(c)), indicating that this stereotactic injection of the virus did not affect the general locomotor activity of these rats. Interestingly, the DE+LV-Con (*P* < 0.01) and WT+LV-Lipin1ShRNA (*P* < 0.05) groups spent less time in the central area than the WT+LV-Con group. In contrast, the DE+LV-Lipin1 group spent significantly more time in the central area than the DE+LV-Con group (*P* < 0.05). Taken together, these results indicate that a reduction in hippocampal CA1 Lipin1 was associated less exploratory activity, suggesting that higher levels of anxiety may be present in these rats.

Results of the NOR test are presented in Figure 3(d). While there were no differences in total exploration times directed to the objects among the four groups, significant differences were present with regard to exploration of the novel versus familiar objects. The WT+LV-Lipin1ShRNA (*P* < 0.05) and DE+LV-Con (*P* < 0.01) groups showed significantly lower DI scores than that of the WT+LV-Con group. In the DE+LV-Lipin1 group, DI scores were significantly greater than that of the DE+LV–Con group (*P* < 0.05). These results indicate that Lipin1 reversed the STZ-induced learning and memory impairments present in DE rats.

In the first two days of the MWM test, no differences were observed among the four groups (Figure 3(e)). On the third and the fourth day, escape latencies of the DE+LV-Con group were significantly increased as compared with those of the WT+LV-Con group (*P* < 0.01). Significant increases in escape latency were also observed in the DE+LV-Con versus the DE+LV-Lipin1 groups on the fourth day (*P* < 0.01). Although escape latencies of the WT+LV-Lipin1ShRNA group were greater than that of the WT+LV-Con group, these differences failed to achieve statistical significance (*P* > 0.05). With regard to the number of platform crossings in the spatial exploration test, the DE+LV-Con and WT+LV-Lipin1ShRNA groups showed significantly fewer crossings as compared with that in the WT+LV-Con group (*P* < 0.05). The number of platform crossings in the DE+LV-Lipin1 group was significantly increased as compared with that of the DE+LV-Con group (*P* < 0.01). These results demonstrate that Lipin1 facilitated the performance of these rats in the MWM test, suggesting that Lipin1 reversed the cognitive deficits observed in these diabetic rats.

### 3.4. Lipin1 within the CA1 Region Affects the Morphological Structure of Dendritic Spines and Synapses

**Figure 4:**
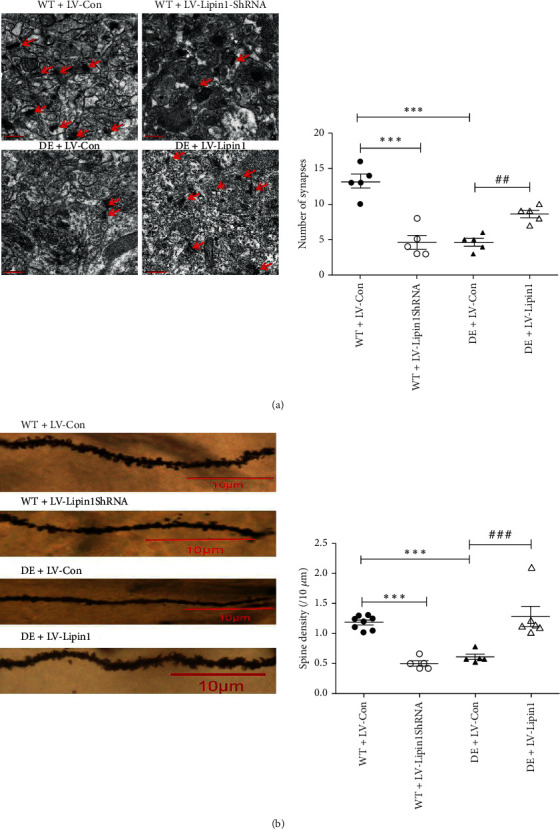
Changes in the morphological structure of dendritic spines and synapses in response to LV injection. (a) Synapses were observed under transmission electron microscopy with arrow indicating location of synapses (scale bar = 0.5 *μ*m). Arrows indicate synapses. (b) Representative Golgi-stained dendritic spine image from the hippocampal CA1 region (scale bar = 10 *μ*m). The images presented were screenshots. The source of these screenshots was shown in the supplementary material Figure S1. Data represent the means ± SEMs, *n* = 5–8 animals per group. ^∗∗∗^*P* < 0.001 versus WT+LV-Con group; ^##^*P* < 0.01, ^###^*P* < 0.001 versus DE+LV-Con group.

Electron microscopy and Golgi staining were used to observe the changes in the neurons within the hippocampal CA1 region of rats. As shown in Figure 4(a), the number of synapses per visual field in the WT+LV-Lipin1ShRNA and the DE+LV-Con groups were significantly less than that in the WT+LV-Con group (*P* < 0.001). Significantly, more synapses were found in the DE+LV-Lipin1 versus DE+LV–Con group (*P* < 0.01). The spine densities, as shown in Figure 4(b), reveal that a significant reduction was present in the WT+LV-Lipin1ShRNA (*P* < 0.001) and the DE+LV-Con (*P* < 0.001) groups as compared with that observed in the WT+LV-Con group. Spine densities in the DE+LV-Lipin1 group were significantly greater than that in the DE+LV-Con group (*P* < 0.001).

### 3.5. Lipin1 Regulates Activity of the PKD/Limk1/Cofilin Signaling Pathway

**Figure 5:**
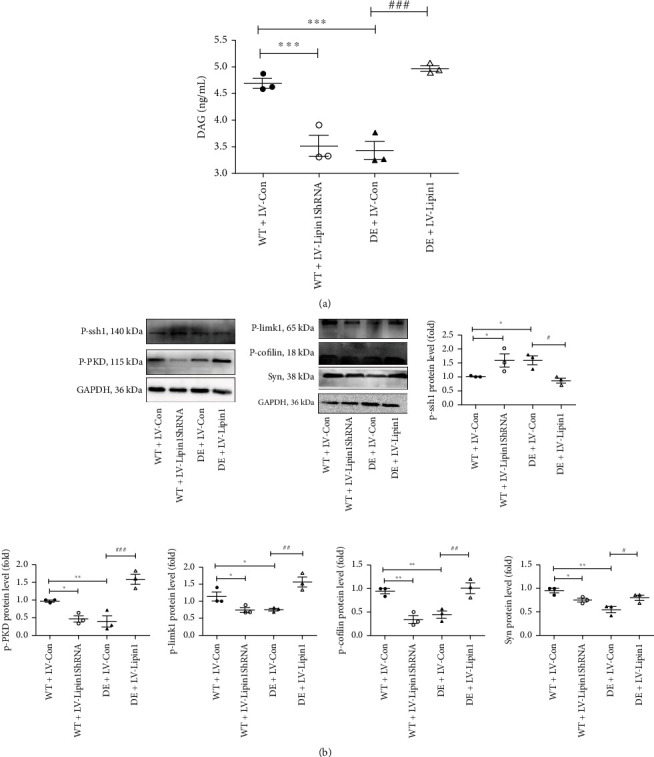
Effect of Lipin1 on activation of DAG, p-ssh1, p-PKD, P-limk1, P-cofilin, and Syn. (a) Levels of DAG were determined using the ELISA kit. (b) Representative Western blot gel results and summary of data showing expression levels of the PKD/Limk1/Cofilin signaling pathway proteins in the CA1 region. ^∗^*P* < 0.05, ^∗∗^*P* < 0.01, versus WT+LV-Con group; ^#^*P* < 0.05, ^##^*P* < 0.01, versus DE+LV-Con group. Results are representative of at least three independent experiments.

Signaling molecule downstream of Lipin1 was detected to further explore the possible neuronal mechanisms of Lipin1 (Figure 5). Levels of DAG in the WT+LV-Lipin1ShRNA and the DE+LV-Con groups were significantly decreased as compared with those in the WT+LV-Con group (*P* < 0.001), whereas DAG levels in the DE+LV-Lipin1 group were significantly increased as compared with those in the DE+LV-Con group (*P* < 0.001, Figure 5(a)). These results are consistent with the changes observed in Lipin1 after virus injection. Signaling pathway proteins of P-Limk1/P-Cofilin were determined using the Western blot assay (Figure 5(b)). As compared with that observed in the WT+LV-Con group, the WT+LV-Lipin1ShRNA and the DE+LV-Con groups showed substantial reductions in P-PKD, P-limk1, P-cofilin, and Syn protein expression levels, whereas these protein levels were significantly increased in the DE+LV-Lipin1 group as compared with those in the DE+LV-Con group. In contrast, protein levels of P-ssh1 in the WT+LV-Lipin1ShRNA and DE+LV-Con groups were increased as compared with the WT+LV-Con group (*P* < 0.05), but were decreased in the DE+LV-Lipin1 as compared with that of the DE+LV-Con group (*P* < 0.05). These results demonstrate that the regulation of these proteins is clearly influenced by Lipin1.

## 4. Discussion

The neurological complications associated with diabetes have attracted considerable attention of late due to their potential in complicating the pathogenesis and therapeutic effects in this condition. Research on the mechanisms of DE have mainly focused on axonal and dendritic changes [15], the prolonged severity associated with deficiencies in insulin [16], abnormal insulin signaling [17, 18], oxidative stress, advanced glycation end-products [19], and synaptic plasticity [20]. Previous results from our laboratory have indicated that increases in Lipin1 content are associated with improvements in nerve conduction velocity and relief of painful neuropathy in DM rats [8]. In the muscle, decreased lipin1 levels and statin treatment staggered to induce autophagy by reducing mTOR, and the ability of autophagy flux will be reduced due to increased PA and decreased activation of PKD, which further leads to impaired autophagy clearance [6]. Moreover, we observed significant impairments in cognitive functions in Lpin1^fld^/J mice [9]. These results suggest that DPN may be related to a decline in Lipin1. In the present study, both *in vitro* and *in vivo* models were employed in an attempt to examine the role and some of the mechanisms of Lipin1 in the regulation of cognitive disorders in DE rats.

PC12 cell, a mature cell line from a rat pheochromocytoma, has been widely used in neurophysiological studies. In particular, these cells have served as a commonly used cellular model for studying the mechanisms underlying glucose neurotoxicity [21–23]. With regard to Lipin1, results from *in vitro* experiments which have shown that Lipin1 concentrations were reduced when PC12 cells were treated with HG and Lipin1 alleviated HG-induced neurotoxicity in these cells. In an attempt to expand upon these findings, we constructed a model of T1DM in rats as achieved with an intraperitoneal injection of STZ as a means to assess these effects of Lipin1 *in vivo*.

Based upon the results obtained with this *in vivo* model, it is clear that Lipin1 affects cognitive functions in rats. The approach used in these experiments involved injections of Lipin1 LVs into the hippocampal CA1 region of these rats. At 14 days after injection, this LV can effectively integrate the transgene and remain expressed for an extended period of time [24]. Following this treatment, the rats were tested in a series of behavioral paradigms including, open field, NOR, and MWM, to assess their activity/anxiety and cognitive functions. The open field test provides a means to assess spontaneous activity, investigation, and anxiety. With this test, animals showing anxiety and depression in this novel environment increase their autonomous activity while they decrease their exploratory activity [25–27]. While the overall general activity of the rats was not different among the groups in this test, the DE group treated with LV-Lipin in the CA1 region showed increased amounts of time spent in the central squares as compared with that observed in DE rats receiving a LV-control injection. When the expression of Lipin1 was decreased in the CA1 region of the WT group, the time spent in the central squares decreased. Accordingly, Lipin1 enhanced the exploration of DE rats, whereas the absence of Lipin1 was associated with a decrease in this exploratory activity. The decreased exploration of rats in the DE and WT+LV-Lipin1ShRNA groups also reflected an increased degree of anxiety in these two groups. The NOR test is based on the nonspatial short-term memory of animals' spontaneous preference for novel objects [28–30]. In the testing period phase of the NOR test, neither hyperglycemia nor the stereotactic injection treatments altered the total amount of time spent investigating the objects, but salient differences were observed among the different treatment groups with regard to their preferences for investigating the familiar versus novel objects. Specifically, we found that Lipin1 treatment prevented deficits produced by hyperglycemia in this test of discriminative memory. Finally, we used the MWM as a means to assess visually related spatial and working memory [13, 31]. Notably, compared with the WT+LV-Con group, the escape latency of WT+LV-Lipin1ShRNA group did not differ. We analyzed the possibility that after the open field and the NOR tests, the rats may have a certain learning ability to compensate. The spatial exploration test results suggested that Lipin1 can improve the spatial learning ability in DE rats. The reduced Lipin1 expression in the CA1 region of WT rats may cause impairment in the spatial learning ability. When collating the results from these behavioral tests, it seems clear that Lipin1 plays a critical role in the behavioral responses examined. In specific, Lipin1 improves cognitive functions and reduces anxiety in DE rats, whereas a reduction in Lipin1 content within the hippocampal CA1 region of WT rats impairs cognitive functions and increases anxiety in these rats.

Next, we examined the effects of these treatments on neuronal structures within the CA1 region with the use of electron microscopy and Golgi staining. We found that the changes in the number of synapses and densities of dendritic spines in each group of rats were consistent with the changes observed in Lipin1 contents and cognitive responses in these rats. That is, perturbations of these neuronal structures were associated with deficits in cognitive functions. Interestingly, in sleep-deprived mice, the number of dendritic spines within the hippocampal CA1 region was selectively reduced, effect which was accompanied with increases in activity of the filamentous actin-severing protein, cofilin [32]. Cofilin, an actin-binding protein commonly found in the cytoskeletal proteins of eukaryotes, promotes cell migration and movement. Activation of cofilin increases f-actin depolymerization and disassembly, causing atrophy and loss of dendritic spines [33–36]. Cofilin is negatively regulated by phosphorylation, inactivated by the Ser3 of limk1, and activated by the dephosphorylation of ssh1 [37, 38]. Through the phosphorylation and inactivation of the SSH at Ser-978, active PKD isoforms markedly increase the phosphorylation of cofilin at Ser-3 to inhibit the actin-driven directed cell migration [39]. During cancer cell migration, SSH1 is identified as a substrate for PKD1-regulation of cofilin activation [40]. With regard to our current results, the Lipin1-related neuronal and cognitive changes within the CA1 region we observed here are similar to that obtained in response to that of cofilin. In addition, we also detected the synthesis of DAG and alterations in the expressions of DAG, p-PKD, p-ssh1, p-limk1, and p-cofilin in these rats. Our findings of decreased levels of DAG and p-PKD within the DE+LV-Con and the WT+LV–Lipin1ShRNA groups were in accord with results from previous reports [6, 9, 41, 42]. Interestingly, as shown in hepatocytes, DAG can produce insulin resistance and contribute to the promotion of type 2 diabetes. As PKD is also involved as an effector of DAG, a deficiency of PKD1 in adipocytes has the effect of increasing insulin sensitivity [43–45]. It has also been reported that stress-induced nascent granule degradation (SINGD) is involved in the development of diabetes as a result of damage to pancreatic beta cells. As PKD is a negative regulator of SINGD, an inhibition of PKD would exacerbate SINGD, thereby contributing to an acceleration of diabetes [46]. These likely differential central and peripheral mechanisms involving complex actions of Lipin1, DAG, and PKD will require further in-depth study. Moreover, the contents of p-limk1 and p-cofilin were decreased in the DE+LV-Con and the WT+LV-Lipin1ShRNA groups, whereas that of p-ssh1 was increased. Accordingly, we also detected changes in synaptophysin, which plays a very important role in regulating activity-dependent synapses [47]. Consistent with the previous results, synaptophysin was decreased in DE rats [48, 49]. In the DE+LV-Lipin1 rats, we observed an increase in synaptophysin, an effect which was associated with improved cognitive functions. A CA1-specific downregulation of Lipin1 in WT rats can have the effects of enhancing cofilin activity and increasing depolymerization and, in this way, reduce dendritic spines, synaptophysin, and cognitive functions. In contrast, an upregulation of Lipin1 in the CA1 of DE rats would suppress cofilin activity, thus improving cognitive functions in DE rats through the PKD/Limk/Cofilin signaling pathway.

## 5. Conclusions

In summary, our results support the hypothesis that sustained hyperglycemia reduces Lipin1 expression within the hippocampal CA1 region to produce a cognitive dysfunction in rats. Neuroprotection of Lipin1 may thus serve as an important neuronal mechanism to ameliorate the cognitive impairments in DE animal models. These neuroprotective effects of Lipin1 are associated with inhibition of the PKD/Limk1/Cofilin signaling pathway. Taken together, these findings suggest that Lipin1 and/or the PKD/Limk1/Cofilin signaling pathway may represent effective protective targets for the development of novel treatments of DE.

## Data Availability

The data used to support the findings of this study are included within the article.

## Abbreviations

BSA: Bovine serum albumin
DAG: Diacylglycerol
DAPI: 4′,6-diamidine-2-phenylidole dihydrochloride
DE: Diabetic encephalopathy
DI: Discrimination index
DM: Diabetes mellitus
DMEM: Dulbecco's modified Eagle's medium
DPN: Diabetic peripheral neuropathy
ELISA: Enzyme-linked immunosorbent assay
FBG: Fasting blood glucose
GAPDH: Glyceraldehyde-3-phosphate dehydrogenase
HG: High glucose
LV: Lentiviral
LVs: Lentiviral vectors
MWM: Morris water maze
mTOR: Mammalian target of rapamycin
NG: Normal glucose
NOR: Novel object recognition
PA: Phosphatidic acid
PFA: Paraformaldehyde
p-limk1: Protein LIM kinase 1
p-PKD: Phosphorylated protein kinase D
qRT-PCR: Quantitative real-time polymerase chain reaction
ShRNA: Short hairpin RNA
STZ: Streptozocin
SINGD: Stress-induced nascent granule degradation.

## Supplementary Materials

Supplementary Materials Figure S1: the source of the Golgi-stained dendritic spine image screenshots.
