# Supplementary material for: Lipin1 Is Involved in the Pathogenesis of Diabetic Encephalopathy through the PKD/Limk/Cofilin Signaling Pathway
Source: Oxid Med Cell Longev. 2020 Oct 16;2020:1723423. doi: 10.1155/2020/1723423 (PMC7586151; doi:10.1155/2020/1723423)
Supplement: Supplementary Materials — Figure S1: the source of the Golgi-stained dendritic spine image screenshots. [file 1723423.f1.docx]

Supplementary Figure S1:

a

WT+LV-con


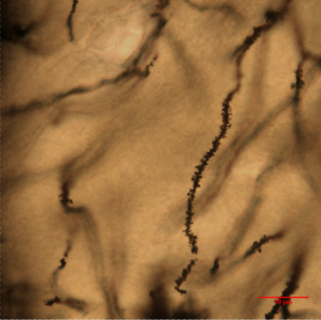


b

WT+LV-Lipin1ShRNA


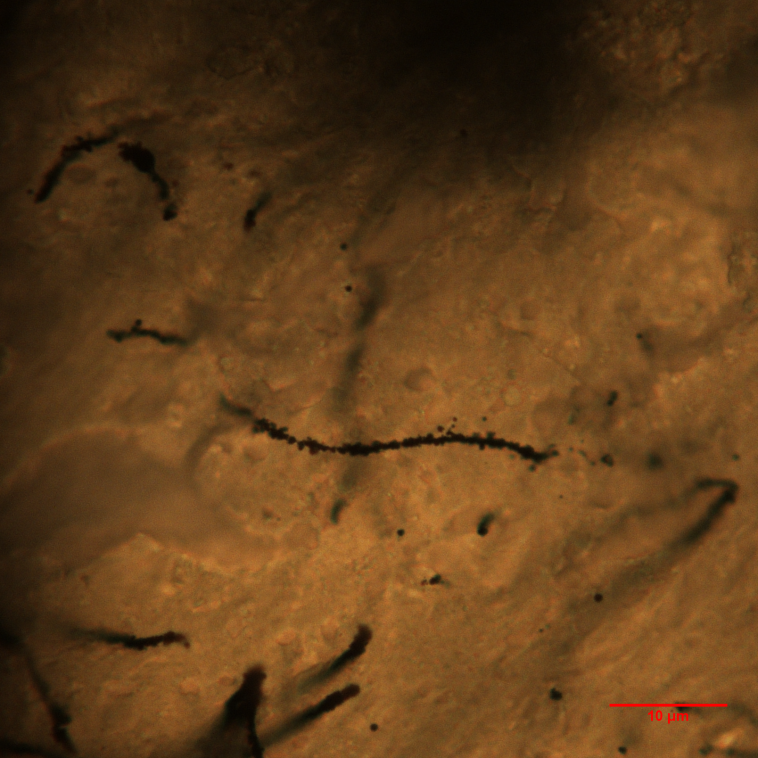


c

DE+LV-con


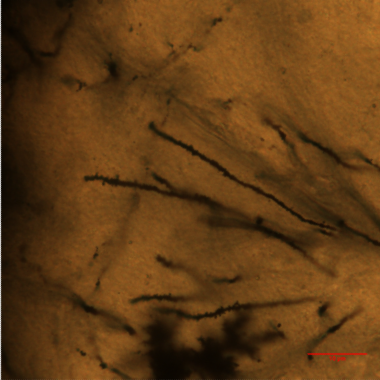


d

DE+LV-Lipin1


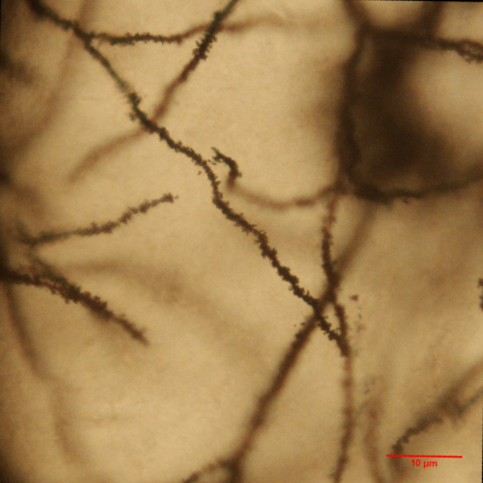


Figure S1: The source of the Golgi stained dendritic spine image screenshots. (a) Representative Golgi stained dendritic spine image from the hippocampal CA1 region in group WT+LV-con. (b) Representative Golgi stained dendritic spine image from the hippocampal CA1 region in group WT+LV-Lipin1ShRNA. (c) Representative Golgi stained dendritic spine image from the hippocampal CA1 regionin group DE+LV-con. (d) Representative Golgi stained dendritic spine image from the hippocampal CA1 region in group DE+LV-Lipin1. (Scale bar = 10 μm).
